# Wearable Active Electrode for sEMG Monitoring Using Two-Channel Brass Dry Electrodes with Reduced Electronics

**DOI:** 10.1155/2020/5950218

**Published:** 2020-07-30

**Authors:** J. Antonio Ruvalcaba, M. I. Gutiérrez, A. Vera, L. Leija

**Affiliations:** ^1^Department of Electrical Engineering, Bioelectronics Section, CINVESTAV-IPN, Zacatenco, Mexico City 07360, Mexico; ^2^CONACYT, Instituto Nacional de Rehabilitación LGII, Subdirección de Investigación Biotecnológica, Mexico City 14389, Mexico

## Abstract

Gel-based electrodes are employed to record sEMG signals for prolonged periods. These signals are used for the control of myoelectric prostheses, clinical analysis, or sports medicine. However, when the gel dries, the electrode-skin impedance increases considerably. Using dry active electrodes (AEs) to compensate variations of impedance is an alternative for long-term recording. This work describes the optimization of the electronic design of a conventional AE by removing the impedance coupling stage and two filters. The proposed work consisted of 5 stages: electrodes, amplification (X250), 2.2 Vdc offset, low-pass filter, and ADC with USART communication. The device did not need the use of electrolytic gel. The measurements of CMRR (96 dB), amplitude of the output sEMG signal (∼1.6 Vp-p), and system bandwidth (15–450 Hz) were performed in order to confirm the reliability of the device as an sEMG signal acquisition system. The SNR values from seven movements performed by eleven volunteers were compared in order to measure the repeatability of the measurements (average 30.32 dB for a wrist flexion). The SNR for wrist flexion measured with the proposed and the commercial system was compared; the values were 49 dB and 60 dB, respectively.

## 1. Introduction

An electromyographic (EMG) signal is the sum of the electrical activity generated by muscle fibers during a contraction. Surface electromyography (sEMG) allows acquiring the EMG signals of the activity of the muscle fibers in a noninvasive way by placing electrodes in contact with the skin. In spite of the volume, the conductor constitutes an important low-pass filter on the EMG signal that intrinsically reduces the measured information [1]; these sEMG signals are still useful for medical diagnosis, prosthetics, rehabilitation devices [2], and sports medicine [3]. However, the amplitude of these signals, which is in the range of 50 *μ*V to 30 mV with a frequency bandwidth from 20 Hz to 450 Hz [4], represents an important concern for developing effective devices aimed at measuring the sEMG signal in long-term recordings without substantial noise and interferences.

Surface electrodes can be categorized into three types: wet, dry contact, and dry noncontact. Current ambulatory biopotential monitoring systems use gel-based (wet) electrodes. Some commercial systems like Shimmer® (Shimmer3 EMG, USA) and Noraxon® (Desktop DTS, USA), developed basically for sEMG diagnosis, not for long-term monitoring, use wet electrodes for the signal recording. Some authors have developed sEMG acquisition systems that use wet Ag/AgCl for signal recording [5]. These systems are highly accurate, but they require skin preparation before recording (washing, alcohol rubbing, or even shaving) and usually require the use of medical tape for fixing the electrodes to the skin. This and a high cost ranging between ∼$17,000 and 20,000 USD make it an impractical recording system for prosthetic applications. However, gel-based systems become unsuitable for ambulatory long-term biopotential monitoring because the gel dries over time, thus reducing the signal integrity and system performance. The use of dry electrodes has been proposed as an alternative for this application [6, 7]; however, when eliminating the electrolyte gel from the electrode, the electric impedance between the electrode and skin increases. This elevates the interference in the system due to cables and movement artifacts [8]. When these dry electrodes are placed on the skin without skin preparation, the impedance increases in magnitude and variability, which makes them susceptible to electromagnetic interference (EMI) [9].

In order to reduce these problems, several metallic materials for dry electrodes of sEMG acquisition have been proposed. Silver [10, 11], copper [12], stainless-steel [10, 13, 14] stainless-steel coated with silicon nitride [15], brass [10, 16], and gold [10, 14] are some of the materials used. Although reports agree that the impedances of dry electrodes are larger than those of wet electrodes, some values are still appropriate for EMG acquisition and analysis. For instance, Anusha et al. [10] and Higashi et al. [11] have reported that silver is a good material for dry electrodes. In another example, Yokus and Jur [17] compared the functionality of dry and wet electrodes during resting and movement, both connected to the same conventional acquisition system; they found that the recordings were similar during the resting phase, but dry electrodes showed higher noise during the motion phase. This problem can be solved by connecting the electrodes directly to a voltage buffer for impedance coupling stage, thus creating an active electrode (AE). The low output impedance of an AE reduces cable motion artifacts, improving the quality of the signal [18]. Many authors use a preamplification circuit as the electrode-skin impedance coupling stage [9, 18, 19], but this increases the number of electronic components on the sensor system, the size of the AE board, and the susceptibility to noise and interferences. Even though buffer-based electrodes reduce power line interference, these systems increase the susceptibility to EMI of the acquisition system. In 2015, the Myo Armband was released [20]. This wireless armband has 8 channels, costs ∼$200 USD, and does not require any skin preparation before recording. However, it has a limited sample rate of 200 kHz with 8-bit precision. Some other approaches in the fabrication of wearable electronics have been presented in the literature, for instance, conductive yarns [4], screen printing [6, 7], photolithography [8], sputtering [10, 11], elastic electronic boards [12], conductive polymers [21], inkjet printing [22], among others. In the literature, there are small-sized, high-cost ASICs for biopotential measurements. An example is the integrated circuit from Texas Instruments ADS1298 which has 8 channels with a programmable gain/bandwidth. The typical CMRR of the system is 115 dB [23]. Also, IMEC develops medical ASICs that address signal quality in connected health applications such as the electrocardiogram (ECG), electroencephalogram (EEG), photoplethysmogram (PPG), galvanic skin response (GSR), electromyogram (EMG), functional near-infrared spectroscopy (fNIRS), bioimpedance, and electrical impedance [24]. The more specific ASIC devices or commands to be manufactured are expensive and, for a study stage, are not suitable to be used during the development and testing period of a new sensor configuration (filtering stages, gain, bandwidths, buffers for impedance coupling, amplification stages, analog conditioning, etc.).

The sEMG signals can be used to continuously activate and control devices such as prostheses and electric orthoses [20]. Another method for reproducing the natural movement of an upper limb by a prosthetic is by implementing inertial measurements [25, 26]. Ruiz-Olaya et al., in 2015, stated that in order to have a good classification and recognition of movement patterns, it is necessary to consider a correct selection of the preprocessing and extraction of characteristics and movement classifier. They found that using sEMG signals for prosthetic control can be difficult due to their time-varying characteristic and that the signals are affected by noise such as the ECG signal or induced electromagnetic interference or by movements [27]. An optimal EMG feature space should contain the following properties: maximum class separation, robust noise, and low computational complexity [28]. There are some time-domain feature extraction methods commonly used for feature extraction from the sEMG signal for movement identification described by other research groups, e.g., maximum amplitude value (MAV), root mean square (RMS) [27], and root mean square successive difference (RMSSD) [29]. Another approach for feature extraction is using symbolic dynamics, which applies analysis from nonlinear systems theory [30]. Oo and Phukpattaranont described techniques for cleaning EMG signals by digital methods, using discrete stationary wavelet transform (DSWT) for eliminating the ECG interference [31]. Zheng and Xu reported the implementation of independent component analysis for removing interferences with minimal distortion of the EMG signal [32].

**Figure 1:**
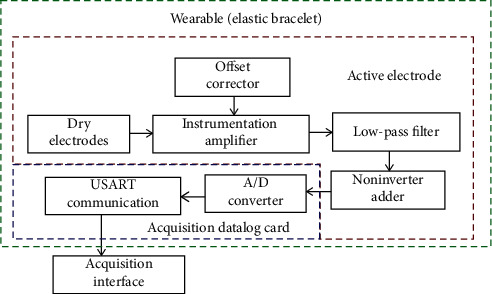
Block diagram of the wearable system for sEMG.

In this paper, a new paradigm for the electronic design of an AE is presented. This proposal consists of eliminating the use of an impedance coupling stage, a high-pass filter stage (for cutting the frequencies from 0–20 Hz), and a band suppressing stage (for cutting the band of 60 Hz). With this optimized AE design, it was not necessary to implement any skin preparations before placing the electrodes. The characteristics of the sensor and the quality of the signals obtained from forearm muscle contractions under two different acquisition protocols were measured in order to demonstrate that this proposal is adequate for mid-term (1-2 hours) sEMG monitoring. Also, the performance of the sensor was compared with a commercial wet electrode sEMG acquisition system. The objective of this work is to demonstrate that the proposed electronic reduction for active electrodes and integration with dry brass electrodes work properly for the acquisition of a wrist movement database and also that it has a low production cost and it is comfortable for the user. Also, the use of brass electrodes does not cause any irritations over the skin in a mid-term use (1-2 hours). The application purpose for the electrodes is to be integrated with the electronic system for sEMG signal acquisition to obtain an active electrode capable of recording good quality signals to avoid further postprocessing to clean the noise and interference signals and thus reduce the computational cost in applications where it is required to record sEMG signals in the real time. In the future, this wearable acquisition system could be used to record wrist movements to help people with prostheses for hand disarticulation amputation level.  Figure 1 presents the block diagram of the parts composing the whole wearable system, including the circuits of the active electrode and the acquisition card (Datalog) used to digitalize the signals. The set of these two cards and the power supply placed over the elastic bracelet completes the wearable for the sEMG signals acquisition.

## 2. Materials and Methods

### 2.1. Active Electrode Design

**Figure 2:**
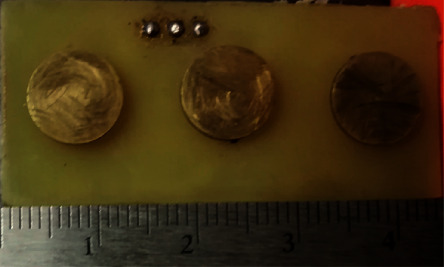
Bottom view of the active electrode. Dry brass electrodes (9 mm diameter and 1.2 mm thick) were placed evenly with an interelectrode distance of 35 mm (differential acquisition); the reference electrode was placed in the middle.

The active electrode proposed here consists of two printed circuit boards (PCBs), one on the top of the other. On the first board, shown in Figure 2, three brass dry electrodes with the shape of a coin were placed following a straight line. The electrodes were soldered directly to the second board, on which the electronic components that corresponded to the system of acquisition of sEMG signals were placed.

#### 2.1.1. Dry Electrodes

Brass dry electrodes were manufactured with a coin shape of 9 mm diameter and a thickness of 1.2 mm. Brass is selected for dry electrode construction because it is a material that is easily machined and could be gold plated; furthermore, it is a material that resists oxidation, specifically for saline liquids [33]. These electrodes were welded to the PCB board with an interelectrode distance of 35 mm in a 3-electrode linear array [23]. The three electrodes were placed evenly spaced following a straight line as seen in Figure 2. The electrodes at the extremes were used for differential acquisition while the electrode in the middle was used as a reference; this middle electrode helped to reduce interferences affecting the acquired signal. After being welded to the PCB, these electrodes were sewed to an elastic band to be worn on the forearm. The elastic band prevents the movement of the electrodes and ensures their contact with skin; this will reduce the noise by movement artifacts. The elastic band also served as an insulator between the brass electrodes and the tracks of the PCB components in order to reduce the probabilities of a short circuit. The integration of the AE with the elastic band makes up the wearable used for the acquisition of the sEMG signals.

#### 2.1.2. Electronic Design

**Figure 3:**
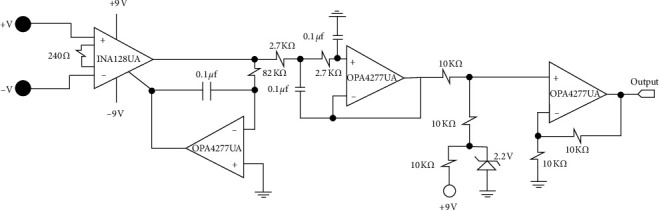
Electronic diagram of the AE.

The brass electrodes were connected directly to the input of an instrumentation amplifier INA128UA (Texas Instruments, USA). In this proposal, the high-pass filter used to remove the frequencies from 0 to 20 Hz, which correspond to the frequencies of motion artifacts [34], was not implemented; instead, the artifacts were automatically reduced with an integrator circuit with a calculated cutoff frequency of 20 Hz. This circuit is also used to eliminate the variable offset of the instrumentation amplifier which is connected to the reference pin of the instrumentation amplifier. The next stage was to implement a unity gain Sallen-Key type low-pass filter with a cutoff frequency of 400 Hz [35]. Digitalization was carried out after adding a 2.2 Vdc offset using a noninverting summing amplifier in order to place the entire signal in the positive region. The circuits corresponding to the low-pass filter, the integrator, and the noninverting summing amplifier were implemented using the OPA4277UA (Texas Instruments, USA). This component has four operational amplifiers which helped reduce the size of the active electrodes. All electronic components were surface mount devices (SMD) so that noise interference was reduced, and electronics were miniaturized in order to obtain a compact active electrode. The electronic diagram for the AE is shown in Figure 3, including the input electrodes and the output signal ready to be delivered to the acquisition card.

### 2.2. Data Collection (Database)

**Figure 4:**
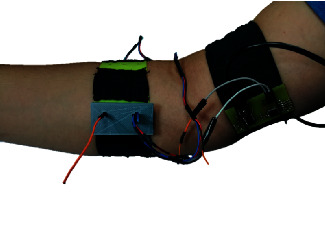
Wearable system with the two AE connected to the ADC and UART-USB transmission card.

The acquisition stage consisted of an ATMEGA328-p microchip for analog/digital conversion (10 bits ADC, 8 channels, and a sampling rate of 1 kHz) and a USART USB connection for transmission of data to the computer. This card was compact (60 × 30 mm), and it was integrated into the wearable together with the sensors. The database was saved in a text file for postprocessing with MATLAB (The MathWorks Inc., USA). The protocols for signal postprocessing depend on the developed test; hence, their descriptions are presented in the respective sections. The electronic board, with the components, placed inside a case with the integrated dry brass electrodes located over the long palmar muscle is shown in Figure 4. In this figure, the Datalog card, the connections with the acquisition system, and the cable for serial communication with the computer are also shown.

### 2.3. Validation Test for the Active Electrode System

Three methods were selected in order to validate the correct functioning of the AE and thus determining that the signals were reliable. The AE was evaluated based on the recommendations for this type of acquisition system [36]. The aim of the first described protocol was the signal comparison with a commercial system; it is just intended to compare the acquisition systems, not the system with the electrodes. The second protocol, between different test subjects, is intended to determine the reproducibility of the whole wearable as an sEMG acquisition system between different subjects.

#### 2.3.1. European SENIAM Recommendations

The adequate functioning of the AE was evaluated based on a series of recommendations of the European SENIAM project (Surface ElectroMyoGraphy for the Non-Invasive Assessment of Muscles) for sEMG sensors. Specifically, the SENIAM project recommended that the common-mode rejection rate (CMRR) should be larger than 96 dB for the proper reduction of common-mode noise. The gain of the sensor must be enough to allow the output signal to reach an adequate range according to the requirements of the used A/D converter. Moreover, the overall lower cutoff frequency should be lower than 10 Hz for EMG spectral analysis, but around 20 Hz for motion analysis; the upper cutoff frequency should be around 500 Hz for most of the applications of EMG [23]. Therefore, the following parameters [36] were determined for the evaluation of the AE proposed:

1. CMRR
2. Gain/amplitude
3. Bandwidth
4. Signal-to-noise ratio (SNR)

#### 2.3.2. SNR for Different Subjects

The repeatability of the measurement system was evaluated with eleven different test subjects: six men and five women, the average age of 27.5 ± 3.4 years. For this purpose, a database of sEMG signals over the long palmar muscle at various wrist movements was created. The signals acquired were processed to determine the signal amplitude and the SNR, for evaluating the quality of the signal obtained with the proposed AE.

Each test subject is given an informed consent form, which they sign, explaining the protocol to follow for taking the signals and the objective of their contribution to the experiment and that the experiment does not cause any discomfort or damage to the test subject skin. During this test, the subject was sitting with their back straight and their forearm resting on a table. An elastic bracelet, which contained two active electrodes (two fully integrated sensors), was placed over the forearm. The sensors were placed in such a way that one was located just above the long palmar muscle and the other one was located over the radial extensor muscle of the carpus. Then, the subject should perform seven individual wrist movements: open hand, close hand, flexion of the wrist, extended wrist, prone wrist, supine wrist, and OK sign (touching the index finger with the thumb, forming a rough circle, and raising the remaining fingers). Each movement was repeated 13 times, following the protocol:

1. Steady state (no movement): from 0 s to 3 s
2. Execution of one of the seven movements: from 3 s to 5 s
3. Steady state (no movement): from 5 s to 6 s
4. Repeat 13 times steps 4 to 5 for the same movement
5. Finish recording

**Figure 5:**
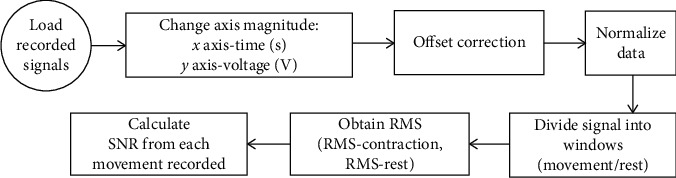
Flow diagram of postprocessing protocol.

The information of the 13 repetitions of each movement was saved in a text file. Each file contained the information of 42 s recorded for the two channels (CH1 and CH2). Each subject performed this protocol twice for each of the seven movements. Therefore, the database was composed of 7 movements, 13 repetitions, 2 times, 11 test subjects, and 2 acquired channels, which yielded a total of 4,004 signals. Each signal was composed of a movement region of 2 s, followed by a stable (rest) region of 1 s. The aforementioned acquisition protocol can be resumed with the flow diagram shown in Figure 5.

Based on Figure 5, the acquisition algorithm loads 14 files (which contain the records of 7 movements; the experiment was carried out twice for each subject). Then, the two channels are separated for each movement and test. Later, the signal amplitude (*y*-axis) is changed from an ASCII value to a voltage value and the acquisition time (0–5 s) is accordingly related to the number of samples (*x*-axis). Before obtaining the SNR, the offset of the signal is removed, and the data is normalized with its own maximum value.

Then, the SNR was calculated for every single signal of the database using equation (1) [37]. This was carried out by determining the root mean square (RMS) value of each signal but separating the two composing regions: *V*_RMS−contraction_, for the movement, and *V*_RMS−stable_, for the stable region. The SNR was obtained with

(1) $$\begin{array}{c} \text{SNR}=20\;\log_{10}\left(\frac{V_{\text{RMS}-\text{contraction}}}{V_{\text{RMS}-\text{stable}}}\right). \end{array}$$

According to [38], an sEMG signal with minimum motion artifacts would have an SNR > 50 dB, while a noisy sEMG signal would have an SNR < 15 dB.

#### 2.3.3. SNR versus Wet Electrode Commercial System

**Figure 6:**
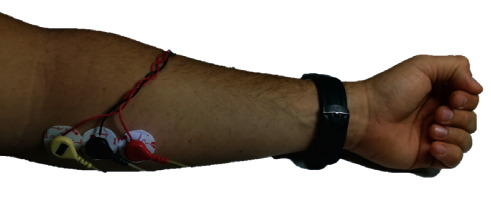
Location of the electrodes on the long palmar muscle for simultaneous connection of the proposed system and the Shimmer commercial system.

As a third validation method of the sensor, we compared the SNR obtained from a signal acquired simultaneously with our system and a commercial system (Shimmer®, Shimmer3 EMG, Boston, USA) using gel-based (wet) Ag/AgCl electrodes. The electrodes were placed in a straight line aligned to the muscle fibers over the long palmar muscle of the right arm of a single test subject (30-year-old, right-handed, healthy male). Three adapters are connected on top of the electrodes, which allowed the Shimmer sensor cable clip to be connected; a cable is connected to each adapter, which was connected to the proposed acquisition system. This configuration is shown in Figure 6. The first and third electrodes were used for differential acquisition and had an interelectrode distance of 30 mm. The second electrode, placed at the center, was the reference electrode. These electrodes were simultaneously connected to a commercial sEMG signal acquisition system Shimmer® (Shimmer3 EMG, Boston USA) and to the acquisition system detailed in this paper. This measurement was carried out in order to compare the magnitude of the signal with the noise ratio in both systems (SNR).

The subject was sitting straight with the forearm resting on a table. Both systems were switched on and started recording at the same time. The subject then performed the seven wrist movements described above. Each movement was stored in an individual file and the next protocol was followed simultaneously for the two systems:

1. Steady state (no movement): from 0 s to 5 s
2. Execute one of the seven movements: from 5 s to 20 s
3. Steady state (no movement): from 20 s to 30 s
4. Finish recording

The signal was split into three sections: rest, movement, and rest. The SNR was determined by using the postprocessing protocol described previously.

## 3. Results

### 3.1. European SENIAM Recommendations

**Figure 7:**
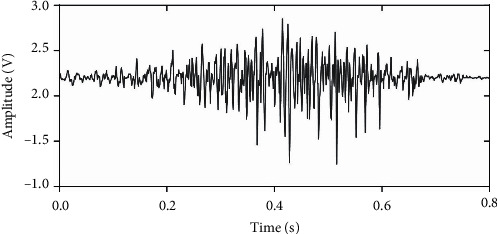
Wrist contraction acquired at the palmar muscle over the forearm using the proposed system.

The electrical parameters were determined based on the recommendations of the European SENIAM project. The CMRR of the system was 96 dB, which was adequate for a wearable sEMG device. The amplitude of a characteristic output during a wrist flection is shown in Figure 7. About this parameter, the SENIAM recommends that the gain of systems for sEMG acquisition should be in the required range determined by the limits and resolution of the used A/D converter; for the purposes of this work, the final overall amplitude of the acquired and conditioned signal had to be smaller than 2 V_dc_. Then, the gain of the amplifier was determined; the calculated gain was 250 and the measured gain was 245; the output signal was between 400 mV_*p-p*_ and 2 V_*p-p*_. A resolution of 10 bits for the analog/digital converter gives a resolution of 4.8 mV per sample; this means that a 10-bit resolution ADC is able to detect the voltage values corresponding to the sEMG signals.

**Figure 8:**
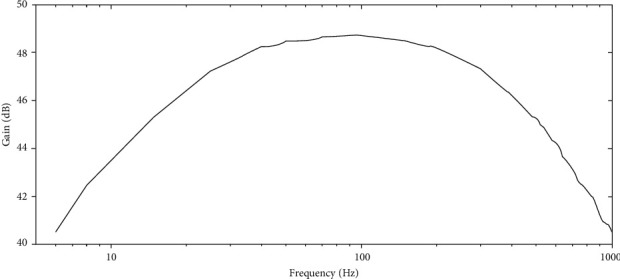
Frequency response of the AE.

The bandwidth of the system was determined based on the graph of Figure 8. This figure shows the frequency response of the complete system after a sine input of 20 mV_*p-p*_ from a signal generator (MSO-X 2022A, Agilent Technologies, USA), using a frequency swept from 4 Hz to 2 kHz. The cutoff frequency for low and high frequencies was taken when the output signal from the system decreased 3 dB in relation to the maximum value of gain. The lower and upper cutoff frequencies of the system were 20 Hz and 450 Hz, respectively. Even though there was no high-pass filter implemented, the cutoff frequency of 20 Hz was produced at the integrator circuit used primarily for offset reduction.

The signals were also analyzed in order to determine the presence of 60 Hz interference. As it was done for the SNR determination, the signal was separated into two parts: the contraction and the resting region. Then, the fast Fourier transform (FFT) of the resting part was determined. The zero-level planar line along the work frequencies (0–450 Hz) [39] indicated that there was no presence of this frequency in the spectrum. The final technical specifications of the active electrode for sEMG, which complies with the recommendations of SENIAM as explained before, are shown in Table 1.

### 3.2. SNR for Different Subjects

**Figure 9:**
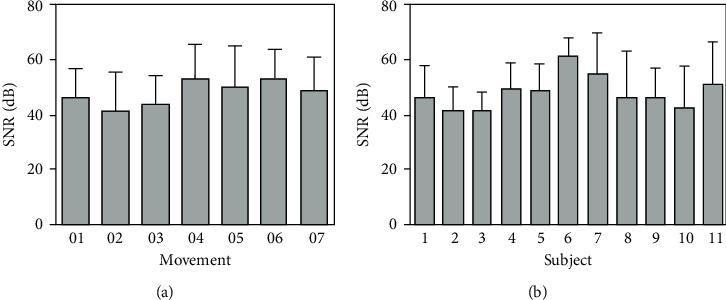
SNR with a standard deviation of signals from CH1 with the protocol for 11 subjects. (a) SNR averaged for each movement among the 11 subjects. (b) SNR averaged for each subject considering the 7 movements. No statistically significant differences were found.

The SNR of the signals measured with the system prosed in this paper was determined for different test subjects when they were performing the seven different movements.  Figure 9 shows the mean SNR of 13 repetitions of each movement/channel/subject which were performed twice. The mean SNR and standard deviation among the subjects, Figure 9(a), were used to evaluate the variations among the different movements used for the test.  Figure 9(b) shows the mean values among the subjects for all the movements. Statistical analysis was carried out in R (3.6.1, R-Project, GNU) using one-way ANOVA with Tukey's HSD post hoc pairwise comparison test. The results among subjects were not statistically different (*p*=0.88, 95% CI [−22.78, 19.58], smallest *p*=0.083 for subjects 2 versus 6), which means that the device works independently to the test subject. Data distribution was assumed normal based on the Shapiro-Wilk normality test with the lowest *p* value of 0.185 for subject 6.

### 3.3. SNR versus Wet Electrode Commercial System

**Figure 10:**
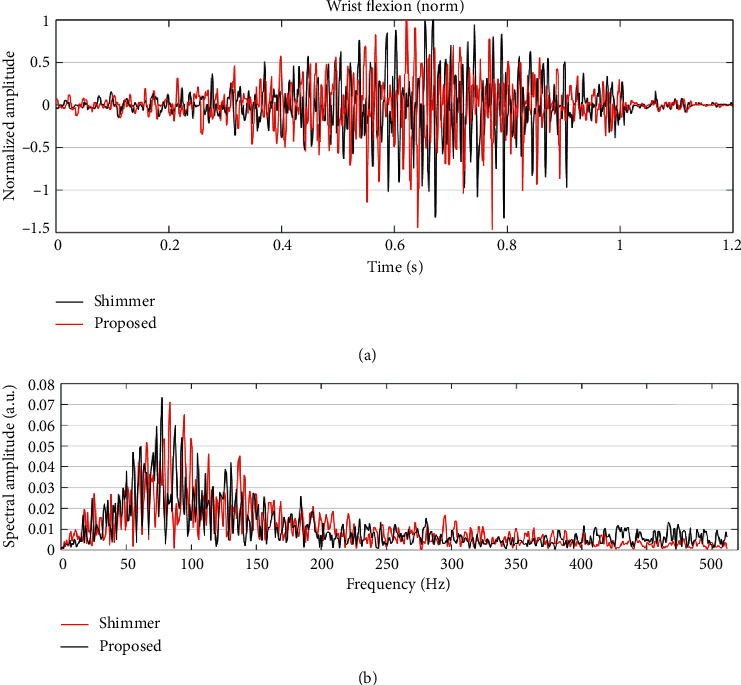
(a) Overlapping of the signals acquired simultaneously by the proposed system and the commercial system of the response of the palmar muscle to a wrist flexion using wet Ag/AgCl electrodes. (b) Overlapping of the spectral components from the signals shown in (a).

In Figure 10(a), we can see the comparison between the signals obtained simultaneously by both systems to the response of the long palmar muscle to a wrist flexion movement, to qualitatively check the similarity and quality between the signals.  Figure 10(b) shows the comparison of the spectral components of the signals in Figure 10(a) where it can be seen, firstly, that both signals have very similar frequencies, and secondly, that the greatest information in both signals is contained between 50 Hz and 150 Hz.

Table 2 shows the SNRs obtained from the signals of each of the 7 movements measured simultaneously with the dry AE proposed here and a commercial system that uses wet electrodes (Shimmer®). Even though the commercial system uses gel-based (wet) electrodes, which permits to record the signals with better quality than systems that use dry electrodes, the SNR values of the AE proposed were quite close to the SNR values of the Shimmer®. The mean SNR and standard deviation (SD) indicated that the performance of the AE proposed here was comparable to that of a system that uses wet electrodes designed for medical diagnosis. To statistically determine the differences among the measurements of the two devices, we performed a two-sided Welch's *t*-test (also known as unequal variances *t*-test), which permits us to analyze if the means of the two data sets (Shimmer and proposed) are significantly different. We chose this *t*-test because we were comparing two independent variables (Shimmer and proposed) with different variances among measurements of each device. The results of this *t*-test indicated that the measurements of both devices are not significantly different (*p*=0.2017) for the data of Table 2.

## 4. Discussion

The sEMG acquisition system proposed in this paper was characterized accordingly to the recommendations of the SENIAM project for sEMG acquisition [36]. The other two validation tests were performed in order to determine the quality of the signal measured under different operation conditions. The active electrode proposed worked accurately with different subjects with repeatable results. The tests performed showed that the AE was a reliable sensor for sEMG long-term recording. The quality of the signals obtained with this device was comparable with that obtained with diagnostic commercial sEMG acquisition systems in terms of gain and SNR.

When measuring the device characteristics, the obtained CMRR had a value that is considered a good EMG acquisition system capable of recording signals with close to zero interferences from the environment according to Merletti and Hermens [40]. This value is adequate for rejecting the common-mode signal in this particular type of acquisition system. With respect to the amplitude of the signal at the output of the sensor, the SENIAM recommends that the gain of the system should be appropriate to have the signal into the input range of the A/D converter; for this work, this maximum positive amplitude was set to 3 V_dc_. Then, after adding the offset to the amplitude of the measured signal, it was appropriate for being digitalized. A characteristic signal measured during a muscle contraction is shown in Figure 7.

The frequency response of the active electrode is shown in Figure 8. The final bandwidth was adequate for this application according to the EMG frequencies [40]. The European SENIAM recommends that the bandwidth of wearable sEMG systems should cover a frequency range from 20 Hz to 400/500 Hz. The integrator circuit allowed cutting off the frequencies smaller than 20 Hz, which are related to movement artifacts, and it allowed eliminating the intrinsic and variable offset coming from the amplifier, as well. Moreover, using the FFT of the resting stage of the measured signal, the absence of the interference frequency of the line at the device (data not shown for simplicity) was confirmed. Because of the use of a simple design implemented with a chip of four operational amplifiers, the size of the system was importantly reduced, and the external interference was negligible.

In order to evaluate the correct operation of the sensor with different test subjects, a series of measurements with three different subjects performing the seven movements were carried out. The SNR was calculated for each signal and the averages for each subject and for each movement of CH1 are shown in Figure 9. These results show that there were movements obtained directly from the long palmar muscle where a signal with better amplitude than others (better SNR) was appreciated, e.g., the signal of wrist supination movement; this affected the quality of the signal and consequently allowed obtaining a higher value of SNR. From Figure 9(a), the maximum SNR corresponds to the supination movement, which was one of the movements with a larger signal. This value contrasted with the minimum for the flexion movement, which was the movement with the lowest signal amplitude. The use of SNR to determine the quality of the response of a device was affected by amplitude variations of the magnitude measured. For instance, it was not possible to compare the signals from CH1 with CH2 since both were obtained from different antagonist muscles; the signal in CH1 was intrinsically larger than the signal in CH2, which produced a larger SNR.

The mean SNR among subjects is compared in Figure 9(b); the differences of these results were not statistically significant according to the value of *p* obtained, which means that the device produced repeatable measurements independently of the subject. However, if the location of the electrodes is not carefully checked, the comparison of results among subjects will not be reliable. Therefore, extra care was taken during the placement of the electrodes in order to avoid any biases in these measurements. Nonetheless, this first set of measurements was used for determining the repeatability of the system when working with different test subjects. The standard deviation of SNRs among subjects was relatively small for movements like hand opening and flexion. The number of test subjects could be a limitation of this study, although based on the small standard deviation among subjects, it is possible to make conclusions about the reliability of the measurements; however, a larger database could be desirable to increase the statistical power. Because the second AE (CH2) placed on the carpal radial extensor muscle is an antagonist to the area recorded with the main sensor (CH1), the intensity of the signal obtained was low; therefore, SNR was also small. For this reason, the second sensor is not being discussed in detail.

Finally, as a third validation method for the AE, the quality of the signal recorded simultaneously with the proposed sensor and with a commercial sEMG signal acquisition system was compared (Table 2). The results of this measurement showed the difference of SNR between both systems for the same acquired signal from the seven different movements. Both systems showed an acceptable SNR according to [38]. The difference of the SNR of the AE proposed and the commercial acquisition system for each one of the seven movements recorded can be determined from Table 2. For some movements, as extension, hand closing, and OK sign, the SNRs were similar in both systems. The mean SNR of the proposal presented here was also acceptable according to literature for a portable system for mid-term sEMG monitoring (wearable) and upper limb rehabilitation.

With the microcontroller used (ATMEGA328-p), there is a limit of 4 acquisition channels with a 1 kHz sampling rate using a 20 MHz crystal. The microcontroller is connected by an FT-232RL to the computer for serial connection. Right now, with two channels, the sampling rate can be changed by reprograming the microcontroller up to 2 kHz. For four channels, the top sampling rate is 1 kHz. To increase the number of channels, it is necessary to use a microcontroller that can work at higher speed like the Arduino Atmel SAM3X8E ARM Cortex-M3 CPU that has an 84 MHz clock. The first results show that it can acquire 8 channels with a sampling frequency of 1 kHz. It is important to mention that another advantage of the proposed wearable with two active electrodes is of low manufacturing cost, with an estimated total cost of ∼$80 USD, including the cost of the boards, the brass electrodes, the batteries, the elastic bracelets, and the components of both the acquisition system and the Datalog card.

## 5. Conclusions

The reduction of electronic components for the optimization of an AE sensor for sEMG signal acquisition system was proposed. Three methods were used to validate the sensor. First, the AE was characterized to comply with the recommendations of the European SENIAM project. Then, the repeatability of the system to work with various test subjects with adequate results was proven. After that, the quality of the acquisition system was compared against a commercial system for sEMG diagnosis; the results showed similar SNRs between the proposed AE and the commercial acquisition system.

Based on these tests, it was proven that the AE with the reduced electronics proposed here worked correctly for the intended application, i.e., for sEMG monitoring for wrist movements recording, and the quality of the signals was comparable to commercial systems and other armbands proposed, described in the introduction, for more demanding applications. Moreover, our device worked immediately after being placed on the forearm of the test subject without any skin preparation needed, delivering a clean sEMG signal with an adequate and handy amplitude (>400 mV_*p-p*_). The system has a low production cost and it is comfortable to use. The wearability of the metal electrode is obtained by the integration of the brass electrodes with the electronic board for the analog processing of the signals (this integration forms the active electrode). In turn, the active electrode is integrated into an elastic bracelet using a sheet of rubber, where the electrodes are fixed, so the sensor can be easily removed. This bracelet or armband (wearable) fixes the active electrode (or EMG sensor) to the skin of the forearm of the test subjects in a comfortable manner. No irritation was observed for the long time periods when the electrodes were used for signal recording. No test subjects showed any signs of skin irritation or any other adverse reaction during these time periods or in subsequent days. However, other methods to characterize the electrodes such as measuring the cytotoxicity of the material are being studied for continuing with the next stages of the project. Also, the AE can be easily removed from the wearable in order to wash the elastic band and clean the system. The quality of the signal allows it to be used for a wide variety of applications from data recording for diagnosis to signal analysis for movement recognition and prosthesis control, for example, for people with amputation at the level of hand disarticulation.`

## Figures and Tables

**Table 1:** Technical specifications of sEMG sensors.

sEMG sensor		
General	# of channels	2
	Resolution	10 bits
	Interface	USB USART
	Sampling rate	1000 Hz
	Power supply	±9 V_dc_
	Size	2.2 cm × 4.7 cm
EMG channels	Input configuration	Single differential
	Input range	0–20 mV
	Gain	245
	High-pass filter	Not required
	Low-pass filter	450 Hz
	Noise level	*μ*V
	CMRR	96 dB
	Input impedance	10^10^‖2Ω‖pF
Additional features	Accessories	Elastic band

**Table 2:** SNR obtained from 7 different wrist movements acquired with the proposed system and the commercial acquisition system Shimmer®.

	Open hand	Close hand	Wrist flexion	Wrist extension	Hand pronation	Hand supination	OK sign	Mean	SD
Acquisition system	SNR (dB)	SNR (dB)	SNR (dB)	SNR (dB)	SNR (dB)	SNR (dB)	SNR (dB)	SNR (dB)	SNR (dB)
Proposed	33.52	49.03	49.88	41.65	43.69	52.10	28.87	42.68	8.73
Shimmer®	49.28	47.49	60.09	41.24	54.87	79.43	31.46	51.98	15.23

## Data Availability

The data used to support the findings of this study are available from the corresponding author upon request.
